# Reactivity of rat bone marrow-derived macrophages to neurotransmitter stimulation in the context of collagen II-induced arthritis

**DOI:** 10.1186/s13075-015-0684-4

**Published:** 2015-06-24

**Authors:** Dominique Muschter, Claudia Göttl, Mandy Vogel, Joachim Grifka, Rainer H. Straub, Susanne Grässel

**Affiliations:** Experimental Orthopedics, Centre for Medical Biotechnology, Biopark I, University of Regensburg, Josef-Engert-Str. 9, 93053 Regensburg, Germany; Department of Orthopedic Surgery, University of Regensburg, Kaiser-Karl V-Allee 3, 93077 Bad Abbach, Germany; Laboratory of Experimental Rheumatology and Neuroendocrine Immunology, Department of Internal Medicine I, University Hospital Regensburg, Franz-Josef-Strauss-Allee 11, 93053 Regensburg, Germany

## Abstract

**Introduction:**

Numerous observations indicate that rheumatoid arthritis (RA) has a bone marrow component. In parallel, local synovial changes depend on neuronal components of the peripheral sympathetic nervous system. Here, we wanted to analyze whether collagen II-induced arthritis (CIA) has an impact on number, adhesion, apoptosis, and proliferation of the macrophage subset of bone marrow cells and how alterations in neurotransmitter microenvironment affect these properties.

**Methods:**

Bone marrow-derived macrophages (BMMs) were isolated from Dark Agouti rats at different stages of CIA, and number, adhesion, caspase 3/7 activity, and proliferation were analyzed in the presence of acetylcholine (ACh), noradrenaline (NA), and vasoactive intestinal peptide (VIP).

**Results:**

Opposed to enhanced CD11b^+^ (cluster of differentiation 11b-positive) and EMR1^+^ (epidermal growth factor-like module-containing mucin-like hormone receptor-like 1-positive) cells, characterizing the macrophage subset, in native bone marrow of rats with acute inflammatory arthritis, we found decreased numbers of CIA macrophages after enrichment and culture in comparison with healthy (control) animals. Adhesion studies revealed significantly reduced attachment to plastic in acute arthritis and collagen type I and fibronectin in chronic arthritis. Additionally, we found a strong reduction in proliferation of BMMs at CIA onset and in the chronic phase of CIA. Apoptosis remained unaffected. Neurotransmitter stimulation profoundly affected proliferation, adhesion, and apoptosis of BMMs from CIA and control rats, depending on disease time point. Cultured BMMs from CIA and control animals expressed neurotransmitter receptors for ACh, VIP and NA, but the expression profile seemed not to be affected by CIA.

**Conclusions:**

Induction of CIA distinctly inhibits proliferation of BMMs in low- and non-inflammatory phases and reduces attachment to plastic at the acute inflammatory arthritis stage and adhesion to collagen I and fibronectin at the chronic stage. Influence of neurotransmitter stimulation on adhesion, apoptosis, and proliferation is altered by CIA depending on disease stage. We suggest an altered reactivity of BMMs to neurotransmitter stimulation caused by CIA and maybe also by aging.

**Electronic supplementary material:**

The online version of this article (doi:10.1186/s13075-015-0684-4) contains supplementary material, which is available to authorized users.

## Introduction

Rheumatoid arthritis (RA) is a systemic autoimmune disorder that is characterized by massive inflammation and destruction of cartilage and bone adjacent to the affected joints. Locally, activated macrophage/monocytes are a major source of pro-inflammatory cytokines like tumor necrosis factor (TNF), interleukin-1beta (IL-1β), IL-6, and chemotactic factors like macrophage inflammatory protein-1beta (MIP-1β) and monocyte chemo-attractant protein-1 (MCP-1), leading to enhanced infiltration of immune cells into the inflamed joint [1, 2]. Owing to pro-osteoclastogenic properties of TNF and IL-1, the microenvironment in inflamed joints provides the prerequisites for a self-fueling, vicious cycle of inflammation and bone destruction induced by macrophage infiltration [3].

The source of macrophage precursors is contained in the hematopoietic stem cell (HSC) niche within the bone marrow of long bones. Several aspects indicate that RA is also a bone marrow disorder [4] (e.g., formation of bone marrow edema [5], a shift in HSC progenitors toward myeloid cells [6], enhanced immuno-senescence of hematopoietic progenitor cells [7], and case-dependent treatment or disease amelioration of RA by bone marrow transplantation (reviewed in [8])).

During RA, changes in local synovial innervation occur and this alters the neurotransmitter composition and may provide a novel regulatory environment for blood- and bone marrow-derived macrophage progenitor cells. During inflammation, anti- and pro-inflammatory functions of the sympathetic nervous system have been described [9]. RA-associated alterations in synovial innervation include the decreased detection of tyrosine hydroxylase (TH)-positive nerve fibers in synovial membrane regions and the appearance of TH-positive cells providing a decentralized source for noradrenaline (NA) [10, 11]. Aside from catecholaminergic signaling, cholinergic signaling is associated mostly with anti-inflammatory effects mediated via the α7 subunit of the nicotinic acetylcholine (ACh) receptor [12]. Cells expressing the α7 ACh receptor were detected in the synovial lining layer of patients with RA and psoriatic arthritis and to a lesser extent in healthy controls [13].

This study intended to analyze whether changes in the local neurotransmitter microenvironment in arthritis affect bone marrow-derived macrophages (BMMs), as inflammatory lesions can penetrate deep into the bone marrow [5]. Immune cells express a variety of neurotransmitter receptors and hence allow extensive modulation by nervous firing [14]. Therefore, we examined whether metabolic properties like number, proliferation, apoptosis, and adhesion of BMMs change in the time course of a collagen II-induced arthritis (CIA) rat model and whether stimulation of cultured BMMs with neurotransmitters NA, ACh, and vasoactive intestinal peptide (VIP) further modulates cell metabolism *in vitro*. The study provides novel information on how progression of CIA alters autonomous macrophage properties and how catecholaminergic and cholinergic/peptidergic neurotransmitters participate in these alterations.

## Methods

### Animals

Female Dark Agouti rats were purchased from Janvier Labs (Le Genest St. Isle, France) at the age of 9 weeks. Animals were housed at four or six animals per cage and were allowed to adapt to animal laboratory conditions for 1 week. Rats were fed standard laboratory chow and water ad libitum and were kept under standard housing conditions in a 12 h light-dark cycle.

### Collagen II-induced arthritis model

All animal experiments were approved by and conducted according to institutional and governmental regulations for experimental animal usage (Ethical Review Committee, Government of the Oberpfalz Az. 54–2532.1-25/13). For arthritis induction, rats were anaesthetized and 300 μg dissolved bovine collagen II (#804001-sol; MD Bioproducts, Egg, Switzerland) emulsified in an equal volume of incomplete Freund adjuvant (Sigma-Aldrich, Taufkirchen, Germany) was intracutaneously injected at the base of the tail. Controls received equal volume of 0.9 % sodium chloride (NaCl) solution (Braun Melsungen AG, Melsungen, Germany). Development of arthritis was monitored by determination of body weight and arthritis score at the respective sampling days. For scoring, each paw was evaluated separately. Three regions were analyzed and one point assigned for signs of inflammation (redness, swelling) occurring in toes/fingers, metatarsus/metacarpus, or ankle. An additional point was given if normal use of the hind limbs was impaired. For front paws, only fingers and metacarpus were evaluated. Altogether, a maximum of 12 points per animal could be assigned (adapted from [9]).

### Isolation of bone marrow macrophages and primary culture

The isolation protocol for rat bone marrow from Ahmed et al. [15] was modified. Shortly, rats were killed 10, 15, 20, and 40 days post-immunization (p. i.) with carbon dioxide (CO_2_), and femur and tibia were collected aseptically. Bones were cut in half and placed in a 1.5 ml reaction tube. Bone marrow was collected by centrifugation (5 min, 5000 × *g*), cells were separated with a 40 μm cell strainer, and erythrocytes were lysed via hypotonic shock in sterile distilled water. For analysis of surface markers CD11b (cluster of differentiation 11b) and EMR1 (epidermal growth factor-like module-containing mucin-like hormone receptor-like 1), native bone marrow cells were collected before erythrocyte lysis. To analyse adhesion, apoptosis, and proliferation, we used an established protocol to enrich BMMs from whole bone marrow by their ability to attach to untreated plastic [16]. Separation via plastic adhesion provides easy and reliable access to the macrophage proportion of bone marrow cells, and the obtained population is further considered to be BMMs. After centrifugation (5 min, 245 × *g*), remaining bone marrow cells were suspended in 20 ml of macrophage medium consisting of alpha-minimum essential medium (α-MEM) with 10 % fetal calf serum (FCS), 2 % glutamate and 1 % antibiotics/antimycotics (Sigma-Aldrich) and 20 ng/ml recombinant rat macrophage colony-stimulating factor (M-CSF) (#400-28; Peprotech, Rocky Hill, NJ, USA). Five milliliters of the suspension was placed in a 100 × 20 mm bacterial petri dish (#430591; Corning, Corning, NY, USA) and cultivated at 37 °C and 5 % CO_2_. After 2 days of pre-culture, plates were washed with phosphate-buffered saline (PBS), and all non-adherent cells in the supernatant were removed. Attached cells, regarded as macrophages, were detached by using 0.02 % ethylene diamine tetraacetic acid (EDTA) in PBS (Sigma-Aldrich), a 5 min incubation period on ice followed by 1 min at −20 °C and a cell scraper. After centrifugation and resuspension in α-MEM, the cell number was determined with a Cedex automated cell counter (Roche Diagnostics GmbH, Mannheim, Germany), and macrophages were seeded at a density of 1.5 × 10^4^ cells/cm^2^ for indicated experiments. Macrophages cultivated in macrophage medium without neurotransmitters are further referred to as non-stimulated cells. Acetylcholine chloride (#A22661), rat/human/porcine vasoactive intestinal peptide (#V6130), and L-(−)-norepinephrine (+)-bitartrate salt monohydrate (#N5785), used for the stimulation experiments, were purchased from Sigma-Aldrich.

### Immunofluorescence staining of neurotransmitter receptors

In total, 10,000 macrophages were seeded into the cavity of an eight-well chamber slide (BD Biosciences, Heidelberg, Germany) and cultured in macrophage medium containing 50 ng/ml recombinant rat soluble receptor activator of NFκB ligand (RANKL) (#400-30; Peprotech) for 5 days with one change of medium after 3 days of culture. After 5 days, cell population (macrophages and osteoclasts) was fixed in 4 % paraformaldehyde for 10 min and washed with PBS. Until staining procedure, cells were stored at 4 °C. Unspecific staining was blocked with 5 % normal goat serum (NGS) (Sigma-Aldrich) in PBS for 20 min at room temperature (RT). Neurotransmitter receptors were detected by using rabbit polyclonal immunoglobuline (Ig) G against rat pituitary adenylate cyclase-activating peptide (PACAP) receptor 1 (1:50, sc-30018, Santa Cruz Biotechnologies, Dallas, TX, USA) and rabbit polyclonal antibody against muscarinic ACh receptor M5 (1:400, ab41171) and rabbit polyclonal antibody against adrenoceptor (AR) β2 (3 μg/ml, ab36956; both Abcam, Cambridge, UK) as well as rabbit polyclonal antibodies against extracellular sections of ARs α1D (1:100, #AAR-019) and α2B (1:100, #AAR-021; Alomone Labs, Jerusalem, Israel). Antibodies were diluted in 1 % NGS/PBS and incubated overnight at 4 °C. After washing, primary antibodies were detected with F(ab′)2 fragment of goat anti-rabbit IgG coupled to Alexa488 (#A11070; Molecular Probes/Life Technologies, Eugene, OR, USA) diluted in 1 % NGS in PBS for 1 h at RT. Nuclei were counterstained with 4′,6-diamidino-2-phenylindole (DAPI), and cells were covered with Fluorescence Mounting Medium from Dako (Hamburg, Germany). Control staining using only the secondary antibody F(ab′)2-fragment revealed no unspecific immunofluorescence. Pre-blocking of the antibodies directed against α1D and α2B ARs with the respective blocking peptide provided by the manufacturer prevented staining and confirmed specificity of the antibody (data not shown).

### Endpoint polymerase chain reaction for neurotransmitter receptors

Total RNA was isolated from macrophages by using the Ambion RNAqueous® Micro total RNA isolation kit (Life Technologies, Carlsbad, CA, USA) in accordance with the instructions of the manufacturer. RNA was reverse-transcribed into complementary deoxyribonucleic acid (cDNA) by using an Affinity Script QPCR cDNA synthesis kit (Agilent Technologies, Santa Clara, CA, USA), and endpoint polymerase chain reaction (PCR) was performed with JumpStart Taq DNA Polymerase (Sigma-Aldrich) and primers for neurotransmitter receptors listed in Additional file 1: Table S1.

### Crystal violet adhesion assay

In total, 5000 macrophages per well were seeded in a 96-well plate and were cultured in macrophage medium without or with 10^−6^ M ACh, 10^−6^ M (β2-agonistic), and 10^−8^ M (α1/2-agonistic) NA or 10^−9^ M VIP (final concentration) for 36 h with no replenishment of neurotransmitter during incubation time. Corning Biocoat™ 96-well plates pre-coated with collagen type I (#354407), fibronectin (#354409), and laminin (#354410) were used for adhesion studies on extracellular matrix (ECM) components (Corning). Non-adherent cells were removed by washing the plates with PBS. Adherent cells were fixed with 1 % glutaraldehyde solution for 30 min and washed with PBS. Fixed cells were stained with 0.02 % crystal violet solution for 15 min at RT and afterwards washed with tap water. The amount of incorporated cristal violet dye is considered to be proportional to the number of cells attached in each well. Incubation in 70 % ethanol for 3 h on a vertical shaker (100 revolutions per minute) dissolved crystal violet out of the cells. Absorbance of ethanol-dissolved crystal violet dye was measured by using a microplate reader (Tecan Group AG, Männedorf, Switzerland) at 600 nm. Differences in absorbance values were correlated with differences in adhesion capacity of cells.

### Caspase 3/7 apoptosis assay

In total, 5000 macrophages per well were seeded in a black 96-well plate with a clear bottom (BD Biosciences, Heidelberg, Germany) and allowed to recover overnight in macrophage medium without neurotransmitters. Cells were synchronized by serum and M-CSF depletion for 24 h. Apoptosis was analyzed by using the APO-ONE Homogenous Caspase 3/7 Assay from Promega (Madison, WI, USA) in accordance with the instructions of the manufacturer. Briefly, serum-free medium was replaced by macrophage medium without or with 10^−6^ M ACh, 10^−6^ M/10^−8^ M NA, or 10^−9^ M VIP (final concentration). Simultaneously, equal volume of caspase 3/7 assay reagent was added and the mixture was incubated for 6–10 h without additional neurotransmitter exchange. The more active caspase 3/7 is present in the cells, the more fluorescent dye is cleaved from the non-fluorescent substrate (Z-DEVD-R110) and differences in emission of varying culture conditions can be measured at 521 nm in a fluorescence plate reader (Tecan Group AG). This information is correlated with apoptotic activity.

### BrdU proliferation assay

With the BrdU (5-bromo-2′-deoxyuridine, thymidine analog) cell proliferation enzyme-linked immunosorbent assay (ELISA) from Roche Diagnostics GmbH (Mannheim, Germany), macrophage proliferation was determined in the presence of ACh, NA, and VIP. Upon addition to cell culture medium, BrdU is constantly incorporated into newly synthesized DNA for a defined period of time. The incorporated amount of BrdU is proportional to the amount of synthesized DNA and allows the comparison of proliferative activity between different conditions after detection with a specific BrdU antibody and colorimetric visualization. For analysis, a total of 5000 macrophages per well were seeded in a 96-well plate and were allowed to recover from seeding procedure overnight. After synchronization by serum and M-CSF depletion for 24 h, cells were cultured 48 h in macrophage medium with BrdU labeling solution without or with 10^−6^ M ACh, 10^−6^ M and 10^−8^ M NA or 10^−9^ M VIP (final concentration) with no replenishment of neurotransmitters during incubation time. After 48 h, cells were processed in accordance with the manufacturer’s instructions, including the use of stop solution, and absorption was measured at 450 nm (reference wavelength of 690 nm) by using a plate reader (Tecan Group AG).

### Flow cytometry

#### Cell cycle analysis

For this analysis, 1.5–2 × 10^6^ macrophages were seeded in a petri dish. After recovery from seeding procedure overnight, macrophages were synchronized by serum and M-CSF deprivation for 48 h to obtain cells synchronized in G_0_/G_1_. Subsequently, cell cycle entry was enabled by the addition of FCS and 20 ng/ml M-CSF, and samples were taken 48 h afterwards. Cell pellets were washed with cold 2 % bovine serum albumin in PBS and fixed in methanol-acetone mixture. Quantitative staining of DNA content with propidium iodide (50 μg/ml for 1 × 10^6^ cells in 500 μl) was performed after RNAse (1 mg/ml; both Sigma-Aldrich) treatment for 1 h at 37 °C. DNA content was analyzed in FACS Calibur (Becton Dickinson, Franklin Lakes, NJ, USA), and data analysis was performed by using the cell cycle tool of FlowJo software (Tree Star, now part of FlowJo, LLC, Ashland, OR, USA).

#### Surface marker and integrin expression analysis

Unspecific staining was blocked with 5 % mouse serum in PBS for 30 min at 4 °C. Native bone marrow cells and cultured BMMs were additionally incubated with 1 μl/1 × 10^6^ cells of purified mouse anti-rat CD32 for 5 min prior to antibody addition to block Fc-mediated adhesion of antibodies to rat FcγIII receptors highly expressed by macrophages (“rat Fc-Block”, #550270; Becton Dickinson). Afterwards, cultured macrophages and native bone marrow cells were stained with antibodies against mouse anti-rat CD11b (phycoerythrin (PE)-coupled, #562105; Becton Dickinson) and uncoupled mouse anti-rat EMR1 (#T-3005; BMA Biomedicals, Augst, Switzerland). Unspecific IgA-PE (#562141) and uncoupled mouse IgG1 (#554121; all Becton Dickinson) were used as isotype controls. After washing, uncoupled antibodies were stained with goat anti-mouse F(ab′)2 fragment Alexa 488-coupled (Life Technologies, Carlsbad, CA, USA, #A-11017) for 30 min at 4 °C.

Integrin expression of cultured BMMs was analyzed by using the following antibodies: Armenian hamster anti-rat CD29 (integrin β1, non-specific for activation status, PE-coupled, #562154; Becton Dickinson) and polyclonal rabbit anti-rat CD51/CD61 (integrin ανβ3, uncoupled, ABIN674784; antibodies-online GmbH, Aachen, Germany). Unspecific Armenian hamster IgM (PE-coupled, #562114; Becton Dickinson) and polyclonal rabbit IgG (uncoupled, ab171870; Abcam) were used as isotype controls. After washing, uncoupled primary antibodies were detected with goat anti-rabbit F(ab′)2 fragment Alexa 488-coupled (Life Technologies, #A-11070) for 30 min at 4 °C.

Stained cells were washed with PBS and suspended in 500 μl of PBS for analysis. Fluorescence was measured with a FACS Calibur (Becton Dickinson). BMM surface marker expression data were analyzed by using FlowJo software (Tree Star). Data for surface marker expression of native bone marrow cells and integrin expression of cultured BMMs were analyzed by using free Software Flowing 2.5.1 (developer Perttu Terho, Turku Centre for Biotechnology, Turku, Finland).

#### Statistical analysis

Statistical analysis was performed by using Prism 4 from GraphPad Software (San Diego, CA, USA). Data are displayed as mean ± standard error of the mean (SEM) or boxplot showing box with median and interquartile range and whiskers from minimum to maximum. Non-parametric Wilcoxon signed rank test for paired observations (neurotransmitter stimulation) was used to test whether individual values were significantly different to control conditions set to 100 %. Variations between effects in controls and CIA groups at the respective time points were compared by using the two-tailed non-parametric Mann-Whitney test.

## Results

### Assessment of experimental arthritis progression and influence on numbers of bone marrow macrophages

To assess induction of experimental arthritis, body weight and arthritis score of immunized (CIA) and control animals (NaCl) were monitored at indicated arthritis time points which represent the course of the disease: 10 days p. i. represents the non-symptomatic phase, 15 days p. i. represents the onset of arthritis with swelling and erythema in single finger joints, 20 days p. i. represents the acute phase of CIA with maximal inflammation in all four extremities, and 40 days p. i. comprises the chronic state with no obvious inflammation but massive destruction of the affected joints (Additional file 2: Figure S1A, B).

Rats immunized with collagen II rapidly lost weight from day 10 p. i., resulting in a maximum weight difference of approximately 20 % compared with controls at day 20 (Additional file 2: Figure S1A). In parallel, the experimental arthritis score rose from approximately 5 points at day 15 to 11.6 points at day 20 and reached a maximum of 12 points until day 40. In contrast, control animals showed no signs of inflammation (Additional file 2: Figure S1B).

**Fig. 1:**
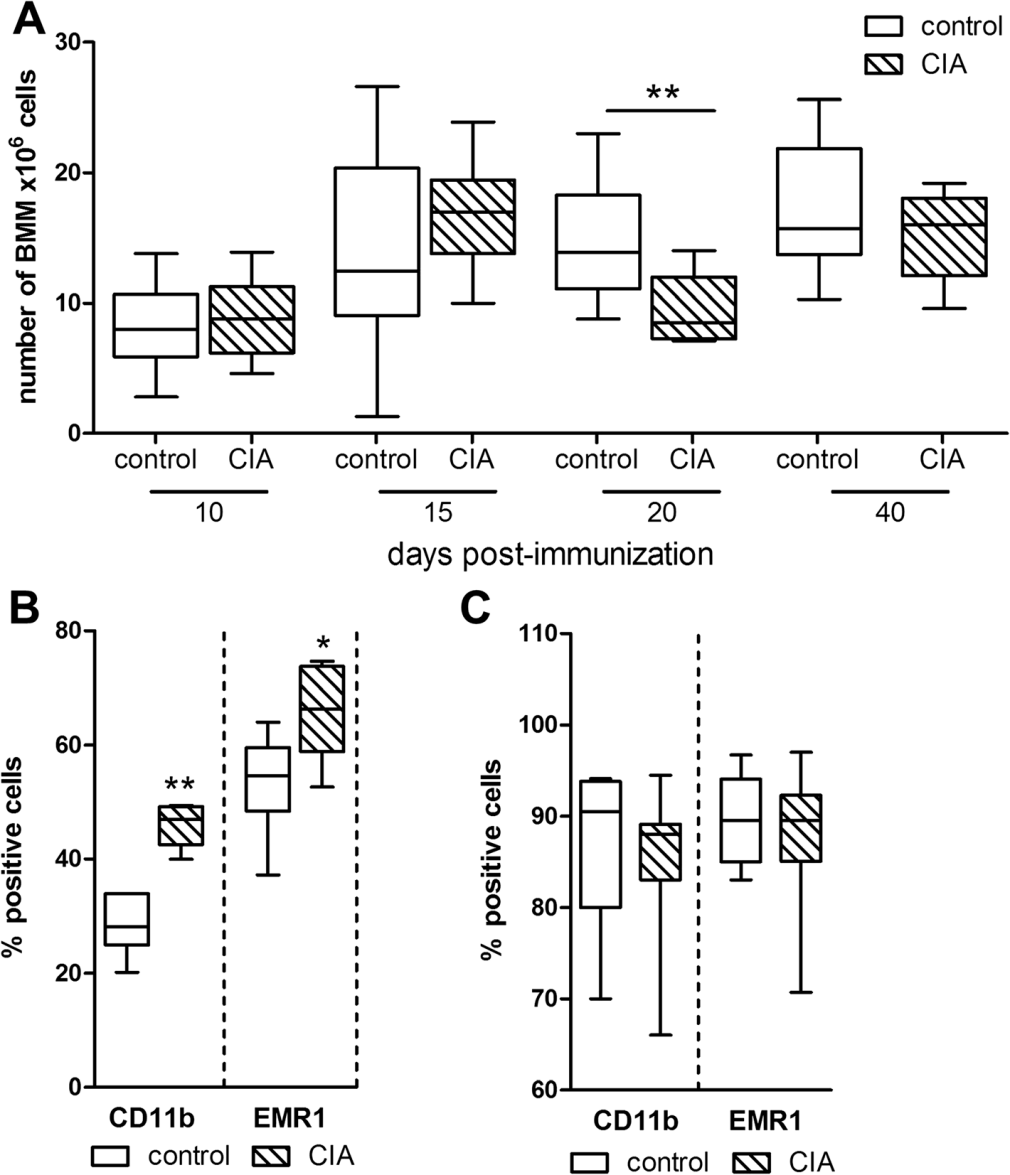
Number of BMMs and analysis of surface marker expression by flow cytometry. **a** Total number of BMMs from control and arthritic rats was determined after 2 days of pre-culture with M-CSF. **P < 0.01. N (control/CIA) = day 10 (9/10), day 15 (10/9), day 20 (10/9), and day 40 (10/9). **b** Native bone marrow cells of control and arthritic rats harvested at the acute disease state (20 days) were characterized by flow cytometry for macrophage-associated markers CD11b and EMR1. *P < 0.05, **P < 0.01. N = 6 for control and CIA. **c** BMMs of control and arthritic animals were harvested after 2 days of pre-culture with M-CSF and characterized by flow cytometry for macrophage-associated surface marker expression of CD11b and EMR1. CD11b: N = 7, EMR1: N = 8 for control and CIA BMMs (sum-up of experimental time points, each N comprises pooled BMMs from 1–3 animals that were either control or CIA). Boxplots show box with median and interquartile range and whiskers from minimum to maximum. *BMM* bone marrow-derived macrophage, *CD11b* cluster of differentiation 11b, *CIA* collagen II-induced arthritis, *EMR1* epidermal growth factor-like module-containing mucine-like hormone receptor-like 1, *M-CSF* macrophage colony-stimulating factor

The total number of isolated BMMs, enriched from bone marrow after 2 days of pre-culture on plastic, was significantly lower in arthritic animals in comparison with controls at 20 days p. i. (Fig. 1a). To analyze whether a defect in the macrophage population of the bone marrow is responsible for this observation, we analyzed native bone marrow cells as well as the cultured enriched BMM population by flow cytometry for markers CD11b (which is expressed on monocytes, granulocytes, and macrophages) and EMR1 (a pan-macrophage marker). Twenty days p. i., corresponding native bone marrow cells from arthritic rats showed a significantly higher percentage of cells positive for macrophage markers CD11b and EMR1 in relation to bone marrow from healthy controls (Fig. 1b). However, we did not detect differences in numbers of CD11b- and EMR1-positive cells between arthritic and control animals in cultured BMMs but did confirm a highly enriched BMM population (up to 90 %) for subsequent analyses (Fig. 1c).

### Macrophages express receptors for acetylcholine, noradrenaline, and vasoactive intestinal peptide

**Fig. 2:**
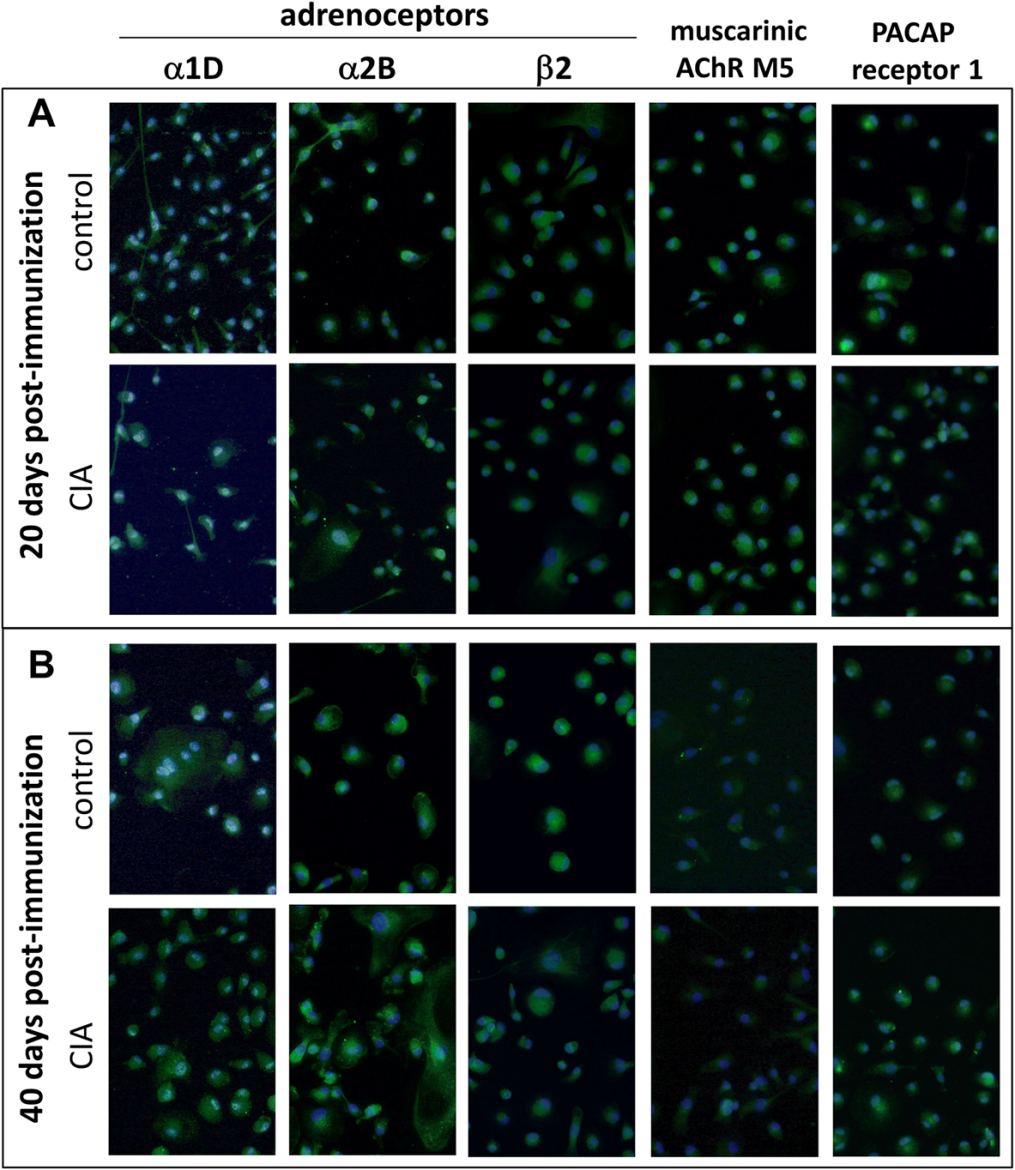
Neurotransmitter receptor profile during course of CIA. Adrenergic receptors alpha 1D, alpha 2B, and beta 2 and muscarinic ACh receptor M5 and the alternative VIP receptor PACAP receptor 1 were stained in BMMs from control and CIA animals 20 (**a**) and 40 (**b**) days post immunization. Paraformaldehyde-fixed cells were stained after 5 days of differentiation in the presence of M-CSF and RANKL, and sections containing only BMMs were photographed. Nuclei were counterstained with DAPI. N = 4 (control and CIA). Magnification 200×. *ACh* acetylcholine, *AChR* acetylcholine receptor, *BMM* bone marrow-derived macrophage, *CIA* collagen II-induced arthritis, *DAPI* 4′,6-diamidino-2-phenylindole, *M-CSF* macrophage colony-stimulating factor, *NA* noradrenaline, *PACAP* pituitary adenylate cyclase-activating peptide, *RANKL* receptor activator of nuclear factor-kappa-B ligand, *VIP* vasoactive intestinal peptide

To assess whether the neurotransmitter reactivity of BMMs is altered during the time course of CIA, we analyzed expression of receptors for ACh, NA, and VIP. We detected mRNA expression of various neurotransmitter receptors (Additional file 3: Figure S2) and decided to further analyze protein expression of ARs α1D, α2B, and β2, PACAP receptor 1 (an alternative VIP receptor), and muscarinic ACh receptor M5 (Fig. 2). Receptors for immunostaining were selected upon searching the current literature for references indicating if any of the receptor subtypes showed altered expression or effector functions in physiological and pathophysiological conditions. Alpha2-ARs play a role in regulation of TNF release from macrophages and can be switched from pro-inflammatory TNF release in healthy conditions to inhibition of TNF release in a chronic constriction injury rat model [17]. This switch would be of interest, as blockade of TNF with therapeutic antibodies seems to play a pivotal role in restoring defective macrophage apoptosis observed in RA [18] or, as presented in a conflicting study, in inhibiting cell migration into inflamed synovial regions of RA patients [19]. Alpha1D-AR mRNA was detected in peripheral blood monocytes from patients with juvenile RA but not in peripheral blood mononuclear cells (PBMCs) of healthy donors [20], indicating a regulatory role for this receptor subtype under inflammatory conditions. For β2-AR, alterations in the sympathetic-to-immune cell signaling during adjuvant-induced arthritis, including immune organ-dependent changes in β2-AR expression, intracellular signaling, and receptor phosphorylation, have been described [21]. Protein analysis of PACAP receptor 1 was included because of its described anti-inflammatory actions representing a promising target for an anti-inflammatory therapy [22]. We detected mRNA for M1, M3, and M5 muscarinic ACh receptors and decided to include only M5 for immunostaining. M3 and M5, but not M1, mRNAs were expressed by PBMCs described by Costa et al. [23]. The rare subtype M5 showed opposite effects in two different studies, making it of specific interest in our study: transfected NIH3T3 cells showed a pro-proliferative response upon M5 receptor agonism [24], whereas a human melanoma cell line, A2058, displayed reduced clonogenic potential using a novel signaling pathway via M5 receptor stimulation [25].

Immunostaining of neurotransmitter receptors appears to be comparable in both BMMs from control and CIA rats, and there appears to be no obvious change related to arthritis progression (20 days Fig. 2a, 40 days Fig. 2b). However, we only qualitatively assessed the staining profile without further quantification.

### Collagen II-induced arthritis reduced macrophage adhesion in acute disease state

**Fig. 3:**
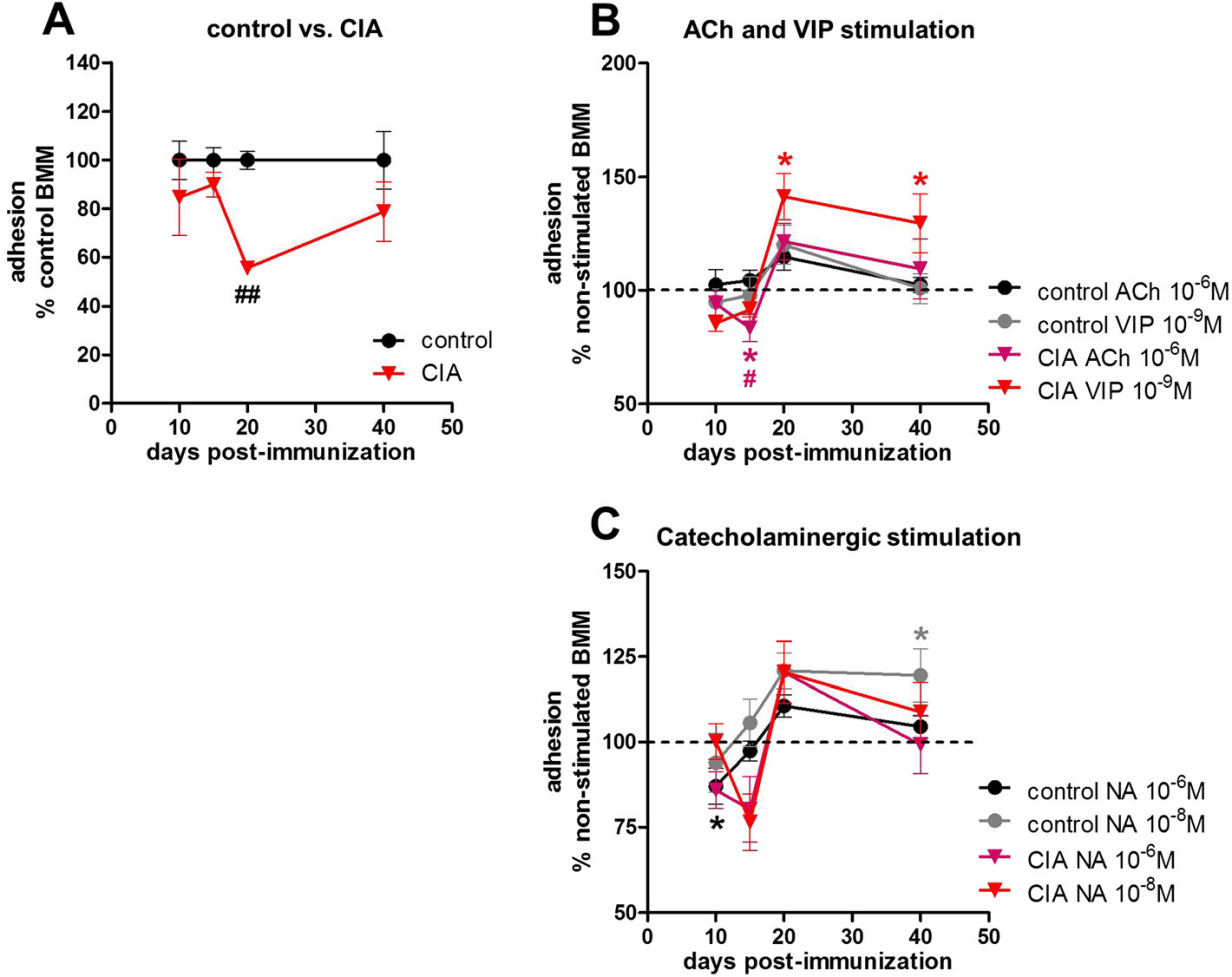
Adhesion of pre-cultured BMMs to plastic surface. Adhesion to plastic surface was measured 36 h after seeding, and pre-cultured BMMs from controls and from CIA animals were compared (**a**). Data are shown as percentage of BMMs from control animals set to 100 %. Influence of stimulation with ACh and VIP on adhesion of BMMs from control and CIA animals is shown as percentage to non-stimulated macrophages (non-stimulated = dotted line) (**b**), and respective results for noradrenergic stimulation are shown under (**c**). N = 6 for control and CIA BMMs and the applied neurotransmitters at each time point. Data are expressed as mean ± standard error of the mean. *P < 0.05 neurotransmitter stimulation versus non-stimulated cells; ^##^ *P* < 0.01 control cells versus CIA cells. *ACh* acetylcholine, *BMM* bone marrow-derived macrophage, *CIA* collagen II-induced arthritis, *NA* noradrenaline, *VIP* vasoactive intestinal peptide

To address the conflicting observations of significantly higher numbers of native monocytes/macrophages in the bone marrow of arthritic animals 20 days p. i. and the corresponding significantly lower number of BMMs harvested after 2 days of pre-culture, we first compared adhesion capacity of cultured BMMs from control and arthritic animals to plastic surfaces (Fig. 3, more detailed data for adhesion are listed in Additional file 4: Table S2A-D). Affirmatively, we detected a significant reduction of adhesion of about 50 % in CIA macrophages compared with control macrophages at day 20 (Fig. 3a).

To analyze whether any of the neurotransmitters has effects on plastic adhesion, we defined the non-stimulated cultures of control and CIA BMMs as 100 % (Fig. 3b and c, dotted line). The neurotransmitter effects on BMMs from control and CIA animals are shown as percentage of the respective non-stimulated culture.

Stimulation with either 10^−6^ M ACh or 10^−9^ M VIP had no significant impact on adhesion of macrophages from control animals (Fig. 3b). On the other hand, in macrophages from arthritic animals, ACh significantly inhibited adhesion on day 15 p. i. compared with non-stimulated CIA BMMs (Fig. 3b). VIP increased adhesion of CIA macrophages on day 20 and 40 p. i. when compared with non-stimulated macrophages (Fig. 3b).

NA (10^−6^ M) (signaling via β-AR) reduced adhesion 10 days p. i. of BMMs from control animals compared with non-stimulated cells. α-AR effects induced by 10^−8^ M NA increased adhesion on day 40 in control macrophages (Fig. 3c). Similar 10^-6^ M NA effects were also observed in CIA macrophages but did not reach significance due to higher data variance (Fig. 3c).

**Fig. 4:**
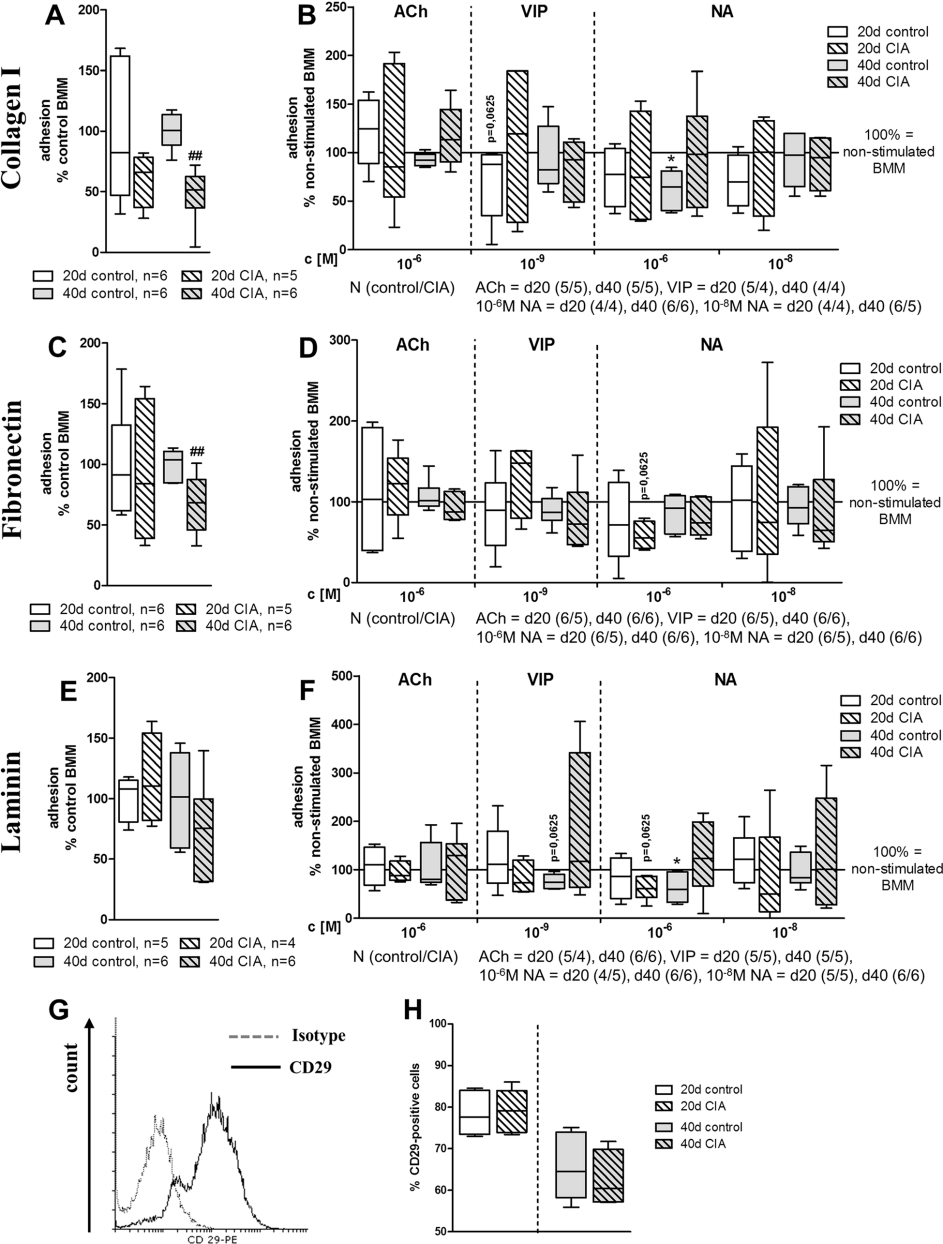
Adhesion of pre-cultured BMMs to extracellular matrix components and expression of integrin CD29. Adhesion to extracellular matrix components collagen type I (**a**, **b**), fibronectin (**c**, **d**), and laminin (**e**, **f**) was measured 36 h after seeding, and cultured BMMs from controls and from CIA animals (**a**, **c**, **e**) 20 and 40 days post immunization were compared. Data are shown as percentage of BMMs from control animals set to 100 %. Influence of stimulation with ACh, VIP, and NA on extracellular matrix adhesion of BMMs from control and CIA animals is shown as percentage to non-stimulated macrophages (100 % = solid line). Results for collagen type I are shown under (**b**), for fibronectin under (**d**), and respective results for laminin are shown under (**f**). *P < 0.05 neurotransmitter stimulation versus non-stimulated cells; ^##^ *P* < 0.01 control cells versus CIA cells. **g**, **h** BMMs were analyzed by flow cytometry after 2 days of pre-culture. BMMs were positive for CD29 (integrin β1, **g**). **h** compares expression levels of CD29 on BMMs from control and arthritic animals 20 and 40 days post immunization. N = 6 for control and CIA at each time point. Boxplots show box with median and interquartile range and whiskers from minimum to maximum. *ACh* acetylcholine, *BMM* bone marrow-derived macrophage, *CIA* collagen II-induced arthritis, *NA* noradrenaline, *VIP* vasoactive intestinal peptide

To determine whether the observed plastic adhesion defect of BMMs from arthritic animals is an artificial *in vitro* cell culture material effect or whether adhesion to ECM compounds is also affected, adhesion to collagen type I, fibronectin, and laminin was analyzed in analogy to tissue culture plastic (Fig. 4). Of all molecules analyzed, adhesion to collagen type I and fibronectin was most noticeably affected, and there was an inhibited adhesion of BMMs from arthritic animals in relation to BMMs from controls by trend 20 days p. i. and significantly 40 days p. i. (Fig. 4a, c). Adhesion to laminin was inhibited 40 days p. i. by trend (Fig. 4e). Analysis of additional neurotransmitter effects on adhesion to ECM molecules revealed no significant effects for ACh and 10^−8^ M NA (Fig. 4b, d, f). 10^−6^ M NA stimulation of BMMs isolated from CIA rats 20 days p. i. showed reduced adhesion to fibronectin (*P* = 0.0625, Fig. 4d) and laminin (*P* = 0.0625, Fig. 4f). In control BMMs isolated 40 days after NaCl treatment, 10^−6^ M NA led to significantly reduced adhesion to collagen type I (Fig. 4b) and laminin (Fig. 4f) in relation to non-stimulated cultures.

By trend, 10^−9^ M VIP inhibited adhesion to collagen type I 20 days after NaCl treatment (*P* = 0.0625, Fig. 4b) and to laminin 40 days after NaCl treatment (*P* = 0.0625, Fig. 4f). Furthermore, we wanted to unravel the underlying molecular reason for the adhesion defect and therefore we analyzed BMM expression of integrins CD29 (integrin β1) and CD51/CD61 (integrin ανβ3)—which critically mediate these cell-matrix interactions—by flow cytometry (Fig. 4g, h). We confirmed expression of CD29 on BMMs (Fig. 4g) but did not detect differences in the percentage of CD29-positive cells between control and arthritic BMMs (Fig. 4h). Integrin CD51/CD61 was not detectable by fluorescence-activated cell sorting (data not shown).

### Caspase 3/7 activity remains unaffected in collagen II-induced arthritis

**Fig. 5:**
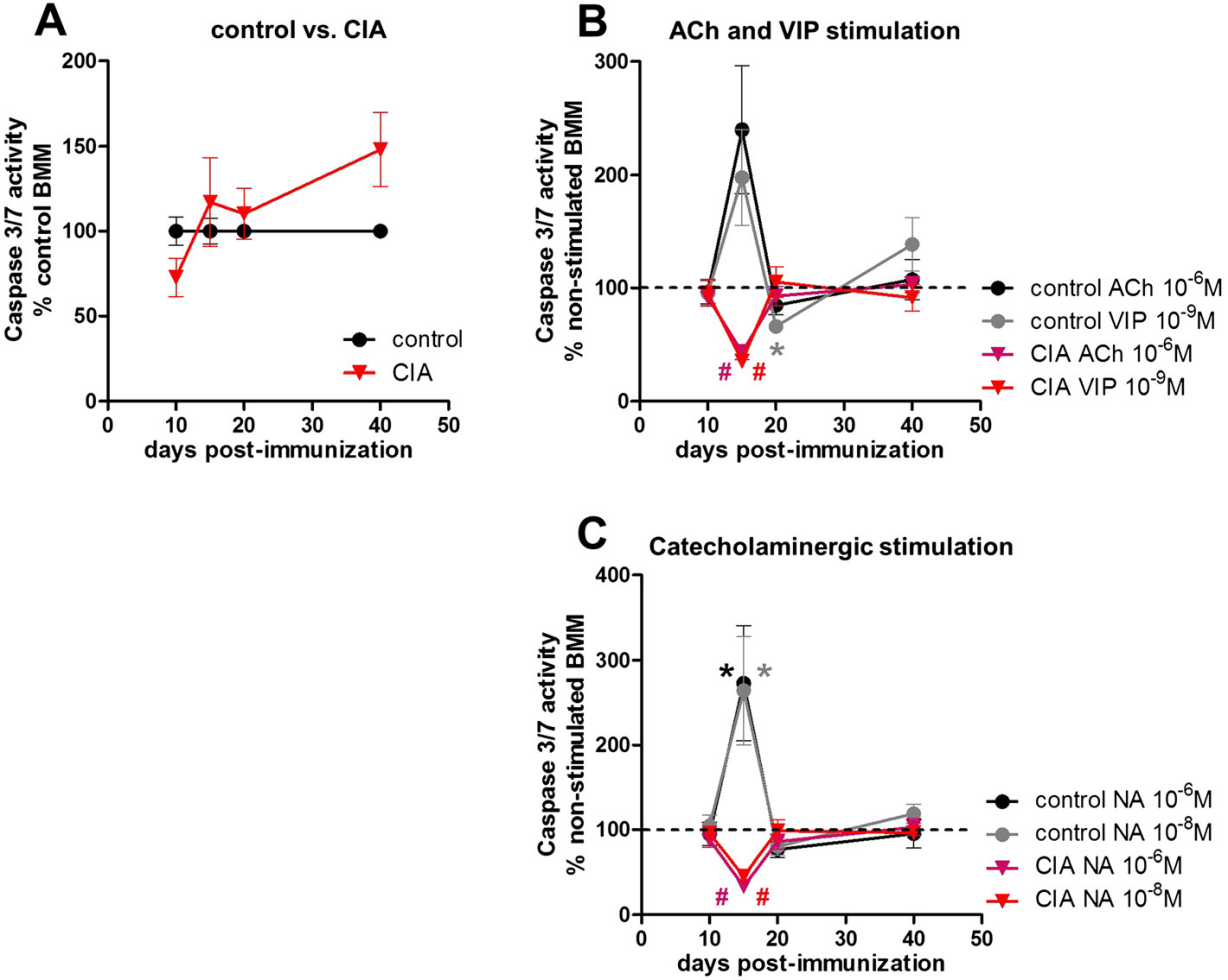
Caspase 3/7 apoptosis assay of pre-cultured BMMs. Pre-cultured BMMs from control and CIA animals which were serum-deprived for 24 h were incubated with caspase 3/7 reagent for 6–10 h. Percentage of apoptosis of CIA BMMs compared with BMMs from controls is presented under (**a**). Influence of ACh and VIP on apoptosis induction in BMMs from control and CIA rats during the time course of arthritis is shown as percentage to non-stimulated macrophages (non-stimulated = dotted line) (**b**). The respective results for noradrenergic stimulation are shown under (**c**). N (control/CIA) = day 10 (9/10), day 15 (12/9), day 20 (10/10), and day 40 (11/12). Neurotransmitter stimulation N (control/CIA) = day 10 (6/6), day 15 (6/3), day 20 (6/6), and day 40 (8/8) for ACh, VIP, and NA (10^−6^ M, 10^−8^ M). Data are expressed as mean ± standard error of the mean. *P < 0.05 neurotransmitter stimulation versus non-stimulated macrophages; ^#^ *P* < 0.05 control cells versus CIA cells. *ACh* acetylcholine, *BMM* bone marrow-derived macrophage, *CIA* collagen II-induced arthritis, *NA* noradrenaline, *VIP* vasoactive intestinal peptide

Changes in apoptotic activity could also explain the reduced number of CIA BMMs (Fig. 5). (For more detailed data, see Additional file 4: Table S2E.)

However, apoptosis remained unaltered when comparing control BMMs with CIA BMMs. At day 40, apoptosis of macrophages from arthritic animals was enhanced but did not reach significance (Fig. 5a).

The effects of neurotransmitter stimulation were analyzed by setting the non-stimulated cultures of control and CIA BMMs as 100 % (Fig. 5b and c, dotted line) and calculating the percentage of the respective neurotransmitter effects on caspase 3/7 activity in control and CIA BMMs.

In control BMMs, α- (10^−8^ M) and β- (10^−6^ M) AR stimulation with NA enhanced apoptosis at day 15 compared with non-stimulated macrophages (Fig. 5c). NA stimulation exerted opposite effects in controls and CIA macrophages, as apoptosis was significantly lower in CIA cells compared with stimulated control BMMs 15 days p. i. (Fig. 5c). 10^−6^ M ACh (*P* = 0.0938) and 10^−9^ M VIP by trend enhanced apoptosis on day 15 in control BMMs (Fig. 5b). Notably, stimulation of CIA macrophages with 10^−9^ M VIP and 10^−6^ M ACh resulted in decreased apoptosis rate at day 15 p. i. in relation to stimulated control BMM cultures (Fig. 5b). After 20 days, VIP effects instead decreased apoptosis rate in controls (Fig. 5b).

### Macrophage proliferation is strongly reduced in collagen II-induced arthritis

**Fig. 6:**
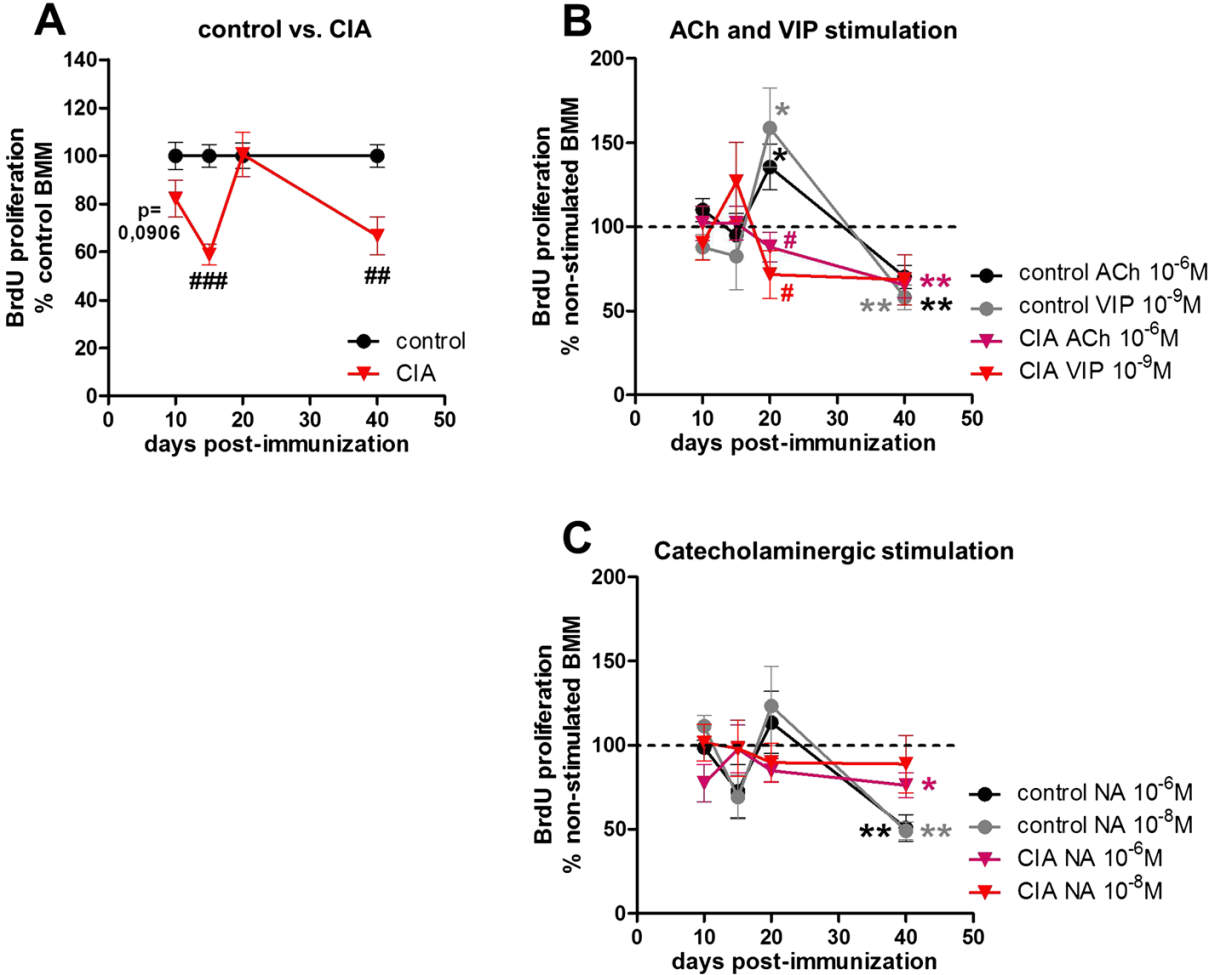
BrdU proliferation assay of pre-cultured BMMs. Pre-cultured BMMs from control and CIA animals which were serum-deprived for 24 h were cultured in the presence of BrdU for 48 h. Percentage of cell proliferation of BMMs from CIA compared with control animals is depicted in (**a**). Influence of ACh and VIP on proliferation of BMMs from control and CIA rats is shown as percentage to non-stimulated macrophages (non-stimulated = dotted line) (**b**). Respective results for noradrenergic stimulation are shown under (**c**). N (control/CIA) = day 10 (11/12), day 15 (12/12), day 20 (12/12), and day 40 (14/14). Neurotransmitter stimulation N (control/CIA) = day 10 (6/6), day 15 (6/6), day 20 (8/8), and day 40 (8/8) for ACh, VIP, and NA (10^−6^ M, 10^−8^ M). Data are expressed as mean ± standard error of the mean. *P < 0.05; **P < 0.01 neurotransmitter stimulation versus non-stimulated cells ^#^ *P* < 0.05; ^##^ *P* < 0.01; ^###^ *P* < 0.001 controls versus CIA. *ACh* acetylcholine, *BMM* bone marrow-derived macrophage, *BrdU* 5-bromo-2′-deoxyuridine, *CIA* collagen II-induced arthritis, *NA* noradrenaline, *VIP* vasoactive intestinal peptide

Another aspect which might contribute to lower macrophage numbers from CIA rats is impaired proliferation (Fig. 6 and Additional file 4: Table S2F).

When comparing proliferative capacity of macrophages from controls with CIA macrophages, a reduction of proliferation in CIA BMMs became noticeable from day 10 p.i. (p=0,0906) and reached significance on days 15 and 40 p.i. (Fig. 6a).

To further dissect the influence of CIA on proliferation, we analyzed the cell cycle distribution of BMMs from control and arthritic animals in the course of CIA. There were no apparent changes in cell cycle distribution at any of the observed arthritis time points (Additional file 5: Figure S3A-D).

The effects of neurotransmitter stimulation were analyzed by setting the non-stimulated cultures of control and CIA BMMs as 100 % (Fig. 6b and c, dotted line) and calculating the percentage of the respective neurotransmitter effects on BrdU incorporation in control and CIA BMMs.

Stimulation of control macrophages with 10^−6^ M ACh and 10^−9^ M VIP resulted in a significant increase on day 20 and a significant decrease in proliferation compared with non-stimulated BMMs on day 40 (Fig. 6b). In CIA BMMs, 10^−6^ M ACh reduced proliferation on day 40 p. i. relative to non-stimulated CIA macrophages. On day 20, ACh and VIP stimulation showed adverse effects between control and CIA macrophages (Fig. 6b), including increased proliferation in control BMMs and reduced proliferation in CIA BMMs.

Stimulation with 10^−8^ M and 10^−6^ M NA reduced proliferation of control macrophages in relation to non-stimulated controls on day 40 (Fig. 6c). In CIA macrophages, stimulation with 10^−6^ M NA resulted in lower proliferative capacity compared with non-stimulated cells 40 days p. i. (Fig. 6c).

## Discussion

Key players in RA inflammatory reactions are macrophages derived from myeloid precursors in the bone marrow. Here, we wanted to analyze how the different CIA stages affect metabolic features like adhesion, apoptosis, and proliferation of BMMs and how changes in local neurotransmitter composition may add to metabolic alterations of macrophages from arthritic animals. The present *in vitro* study allowed us to dissect the neurotransmitter influence from *in vivo* effects like co-transmitter signaling and high levels of pro-inflammatory cytokines (i.e. TNF and IL-1β).

To study macrophage characteristics, cells were enriched from whole bone marrow preparations by their ability to attach to untreated plastic surfaces [16]. Comparing the different stages of CIA, we observed a noticeable decline in macrophage numbers after 2 days of pre-culture on plastic surfaces from bone marrow isolated at the acute disease stage with high-grade joint inflammation. To elucidate whether reduced monocyte/macrophage numbers in the native bone marrow are the underlying cause, we analyzed the proportion of CD11b-positive and EMR1-positive (rat homolog to murine F4/80) cells in controls and animals with acute CIA. Strikingly, both markers were significantly elevated in the bone marrow cell suspension of rats with acute arthritis. Consistently, although Nishida et al. [26] found markedly reduced total bone marrow cell numbers in a murine CIA model which is similar to patients with RA [27], the proportion of cells from the myeloid lineage was found to be elevated in murine arthritis models [6, 28]. The unsettled question remained, what has caused the profound loss of macrophage numbers from acutely arthritic animals after the 2-day enrichment phase on plastic culture dishes?

Contributing to this observation might be the fact that in our *in vitro* system we observed a strong reduction in adherence of macrophages to plastic surfaces at the acute arthritis stage, which might cause the lower BMM and precursor cell numbers. Changes in plastic adherence have been shown for macrophages from patients with systemic lupus erythematosus and RA, but results could have been influenced by corticosteroid treatment [29]. We show that in the CIA rat model this effect is independent of treatment and therefore may represent a metabolic alteration caused solely by inflammation.

In addition to observing this CIA effect, we observed clear alterations in plastic adhesion in the presence of NA, ACh, and VIP. By qualitative analysis of gene expression, we ensured that BMMs express a high range of receptors for NA, ACh, and VIP. Immunostaining of selected receptors showed no qualitative differences in receptor expression by CIA or control BMMs, and the expression of various receptors for specific neurotransmitters might result in integrative effects for each of the neurotransmitters. In case of adhesion to plastic, NA effects were stronger in BMMs isolated from NaCl-treated control rats, with dose-dependent reduction of adhesion (10^−6^ M) 15 days after NaCl treatment and enhancement (10^−8^ M) after 40 days. This timely and possibly age-dependent effect of NA influence on plastic adhesion was also observed by Ortega et al. using peritoneal macrophages [30]. We further report an influence of ACh and VIP on plastic adhesion of BMMs from CIA rats, which has not been investigated before. At disease onset, ACh inhibited adhesion whereas in acute and chronic stages VIP effects became more effective and strongly increased plastic adhesion of CIA BMMs. Although there are no direct data available for macrophages, Reardon et al. found a decreased expression of adhesion molecules on endothelial cells after ACh stimulation, inhibiting neutrophil recruitment to inflammatory regions [31]. This effect was dependent on muscarinic receptors. In line with our data, de la Fuente et al. observed that stimulation of peritoneal macrophages with 10^−9^ M VIP clearly enhanced adhesion [32]. A more detailed analysis of BMM adhesion to specific components of ECM would allow conclusions involving *in vivo* mechanisms as macrophages migrate via anchorage to these macromolecules. Therefore, attachment studies on collagen type I, fibronectin, and laminin were conducted. Studies with peripheral blood monocytes [33] and synovial fibroblasts [34] derived from patients with RA revealed increased integrin expression and enhanced attachment to ECM molecules. However, we observed a noticeable reduction in adhesion to type I collagen and fibronectin at the chronic disease state. Data for the acute arthritis time-point did not reach significance possibly due to small sample sizes. Attachment to laminin was inhibited by CIA by trend in the chronic phase. In contrast to plastic adherence, adhesion to collagen I and laminin was inhibited in control BMMs in the presence of 10^−6^ M NA 40 days after NaCl treatment. In BMMs derived from rats with acute CIA, 10^−6^ M NA instead reduced adhesion to fibronectin and laminin. 10^−9^ M VIP tended to reduce adhesion of control BMMs to collagen type I and laminin 20 and 40 days after NaCl treatment, respectively. ACh and 10^−8^ M NA failed to induce changes in adhesion to any of the analyzed macromolecules (collagen I, laminin, or fibronectin). Altogether, we found an intrinsic defect in adhesion to plastic, collagen I, and fibronectin and partly to laminin that was caused by CIA and, in regard to macromolecules of the ECM, would provide a handicap to enter sites of inflammation. NA effects observed under physiological conditions are dampened in arthritis. ACh and VIP influences on plastic adherence emerge only under arthritic conditions, and VIP effects on adhesion to laminin and collagen type I affect only BMMs from healthy rats, indicating a changing neurotransmitter response concerning adhesion processes to different substrates caused by unknown, CIA-associated mechanisms.

Concomitant flow cytometry studies of CD29 (integrin β1) expression revealed no differences for BMMs from arthritic rats. The activation status of CD29 was not distinguishable with the applied antibody; hence, we do not know whether CIA induced higher activity or abolished any activated state. CD51/CD61 (integrin ανβ3), another integrin involved in ECM adhesion, was not detectable in our culture set-up.

A major observation of this study included changes in BMM proliferative capacity which occurred as early as 10 days p. i. (by trend) and persisted until the chronic state with surprisingly little differences at the acute arthritis stage. Implicating the decreased adhesion of CIA BMMs at day 20 p. i., we presume a higher macrophage proliferative capacity at the acute arthritis stage. Enhanced proliferation in acute inflammatory reactions reflects a strong requirement for immune cells to resolve inflammation, and a local proliferative burst has been described for macrophages under acute inflammatory conditions [35]. On the other hand, RA profoundly impairs proliferative capacity of CD34^+^ hematopoietic macrophage progenitor cells by inducing early immuno-senescence in the HSC compartment [36]. A reduced proliferative responsiveness to mitotic stimulation has also been reported for spleen cells and peripheral blood monocytes isolated from rats with adjuvant-induced arthritis as early as 4 days after induction [37]. Impaired proliferation reduces the HSC pool, but high levels of local and systemic pro-inflammatory and pro-proliferative factors at the acute arthritis stage may overcome this mechanism and may lead to exhaustion of precursor pools in chronic conditions. 10^−6^ and 10^−8^ M NA strongly decreased proliferation in control BMMs 40 days after NaCl treatment, but only 10^−6^ M affected CIA BMMs with a lower effect size. Similarly, as shown for cell culture assays, high- and low-dose NA—acting via β- and α-ARs, respectively—decreased proliferation of BMMs [38]. In contrast, stimulation with VIP and ACh enhanced proliferation in BMMs from controls, whereas the effect was abrogated in CIA cells at the acute arthritis time point. At the chronic time point, ACh and VIP blocked proliferation of BMMs from both controls and arthritic animals. ACh and VIP effects have been demonstrated to be predominantly anti-inflammatory [39, 40] and this is in line with our data. The anti-inflammatory effects of ACh have been ascribed mainly to signaling through the α7 receptor subunit of the nicotinic ACh receptor (“cholinergic anti-inflammatory reflex”) [41]. Surprisingly, we failed to detect gene and protein expression for α7 nAChR and therefore cannot assign observed ACh effects to that specific receptor. Instead, we detected muscarinic M1, M3, and M5 receptor mRNA, receptor subtypes sharing the same signaling cascade [42]. M5 receptor agonism induced a pro-proliferative response in cell culture experiments using transfected NIH3T3 cells [24]. Interestingly, a human melanoma cell line, A2058, displayed reduced clonogenic potential using a novel signaling pathway via M5 receptor stimulation [25] and this might explain the opposite ACh effects in acute and chronic CIA. VIP stimulation was shown to inhibit proliferation of HSC progenitor cells through VIP receptor 1 [43].

Low-dose NA, also expected to be caused by retraction of TH-positive nerve fibres in vivo [10], did not suppress proliferation in CIA BMMs. Possibly, ACh and VIP compensate for this CIA effect in the chronic arthritis stage.

In contrast to proliferation, insufficient apoptosis of macrophages, which may be one of the underlying causes for longtime disease persistence, seems to be locally restricted to the synovial tissue [44] as the results of the present study show no apparent changes in BMM apoptosis. Notably, for caspase 3/7-mediated apoptosis, we observed opposite effects of NA with profound induction of apoptosis in BMMs from controls and protection in CIA. This was also true for ACh and VIP. These data implicate that, at onset, CIA, via unknown mechanisms, preferably protects BMMs from neurotransmitter-induced apoptosis. In cell culture assays, NA effects mediated via β- and α-ARs, respectively, would dose-dependently induce apoptosis in lymphocytes and a macrophage cell line [45, 46]. VIP effects were opposite but corroborate the contrary observations in CIA and control BMMs: VIP strongly enhanced apoptotic caspase 3/7 activity in neutrophils from healthy volunteers [47] but was also shown to rescue murine acinar cells from TNF-induced apoptosis [48]. Instead, M3 and M5 muscarinic ACh receptors have neuroprotective effects by preventing apoptosis [49].

A striking observation made in this study was the fact that not only BMMs from CIA rats showed differential reactivity to neurotransmitter stimulation over time, related to different disease stages, but also BMMs derived from controls. Although we did not analyze this phenomenon more in depth, we suspect aging effects to be the underlying cause, as this has been shown for NA effects on plastic adhesion of peritoneal macrophages by Ortega et al. [30].

## Conclusions

Here, we provide strong evidence that collagen-induced arthritis alters intrinsic metabolic capacities of macrophages isolated from rat bone marrow and their reactivity to neurotransmitter stimulation with NA, ACh, and VIP. Starting early after immunization, CIA initiates mechanisms that lead to impaired proliferative activity exclusively at low and non-inflammatory stages. Highly inflammatory conditions may override the underlying effects and restore or even enhance proliferation. Anti-proliferative effects of low-dose NA were abrogated in chronic arthritis, but altered reactivity to ACh and VIP compensated for this effect and instead reduced BMM proliferation, emphasizing the anti-inflammatory effects of these neurotransmitters.

Pro-inflammatory conditions instead cause a strongly reduced plastic adhesion of macrophages evoking alterations in *in vivo*-generated macrophage numbers. Additionally, defective adhesion to type I collagen and fibronectin was observed in chronic arthritis and might represent an actual mechanism to hinder adhesion to ECM and to prevent new inflammatory processes in the joints of rats with chronic arthritis. Noradrenergic inhibition of adhesion to collagen type I and laminin was effective only in BMMs from healthy rats. An anergic state of CIA BMMs toward NA-mediated inhibition of adhesion to ECM macromolecules would, in opposition to observed CIA effects, promote stronger infiltration.

From that we can conclude that BMMs are differentially affected in their metabolic activity depending on disease stage and the associated inflammatory status. Clearly, all neurotransmitters investigated had strong adverse effects in control and CIA macrophages, but modulation occurred in a time- and assay-dependent manner. We suggest that collagen-induced arthritis causes an altered reactivity to neurotransmitter stimulation which has compensatory functions in BMMs.

## Additional files

Additional file 1: Table S1. Primer sequences for endpoint polymerase chain reaction (PCR). *bp* base pairs. Additional file 2: Figure S1. Collagen type II-induced arthritis (CIA). Successful CIA induction was verified by monitoring body weight (**a**) and arthritis score (**b**) of arthritic and control animals 10 (*n* = 40), 15 (*n* = 30), 20 (*n* = 20), and 40 (*n* = 10) days post immunization. Data are expressed as mean ± standard error of the mean. ***P* < 0.01, ****P* < 0.001. Additional file 3: Figure S2. Endpoint polymerase chain reaction for neurotransmitter receptors. Gene expression of neurotransmitter receptors for ACh, NA, and VIP was analyzed by endpoint polymerase chain reaction by using RNA isolated from BMM of controls 10 days (**a**) and 20 days (**b**) following sodium chloride treatment. N = 1. *AR* adrenoceptors, *BMM* bone marrow-derived macrophage, *bp* base pairs, *M1-M5* muscarinic acetylcholine receptor, *NA* noradrenaline, *nAChR* nicotinic acetylcholine receptor, *PACAPR1* pituitary adenylate cyclase-activating peptide receptor 1, *VIPR1-2* vasoactive intestinal peptide receptors 1 and 2. Additional file 4: Table S2. Data summary for adhesion, apoptosis, and proliferation assay. Table S2 summarizes data for adhesion assay (**a**-**d**), caspase 3/7 assay (**e**), and proliferation assay (**f**). Non-stimulated CIA data are shown as percentage to BMM from control (100 %). Effects of neurotransmitter stimulation are presented as percentage to the respective non-stimulated CIA and control BMM (100 %). Data are expressed as mean ± standard error of the mean. **P* < 0.05 neurotransmitter stimulation versus non-stimulated cells; ^#^ *P* < 0.05; ^##^ *P* < 0.01; ^###^ *P* < 0.001 control cells versus CIA cells. *ACh* acetylcholine, *BMM* bone marrow-derived macrophage, *CIA* collagen II-induced arthritis, *NA* noradrenaline, *VIP* vasoactive intestinal peptide. Additional file 5: Figure S3. Cell cycle analysis. G_0_/G_1_ phase BMMs from control and CIA rats were cultured in the presence of fetal calf serum and macrophage colony-stimulating factor for 48 h and cell cycle distribution was analyzed by measurement of nucleic propidium iodide content. The graph compares the frequency of BMMs from control and CIA animals distributed in cell cycle phases G_0_/G_1_, S, and G_2_/M. Results for 10 days p. i. are presented under (**a**), 15 days p. i. under (**b**), 20 days p. i. under (**c**) and 40 days p. i. under (**d**). N = 6 for control and CIA at each time point. *BMM* bone marrow-derived macrophage, *CIA* collagen II-induced arthritis, *p. i.* post-immunization.

## Abbreviations

ACh: Acetylcholine
AR: Adrenoceptor
BMM: Bone marrow-derived macrophage
BrdU: 5-bromo-2′-deoxyuridine
CD: Cluster of differentiation
cDNA: Complementary deoxyribonucleic acid
CIA: Collagen type II-induced arthritis
CO_2_: Carbon dioxide
ECM: Extracellular matrix
EMR-1: Epidermal growth factor-like module-containing mucin-like hormone receptor- like 1
FCS: Fetal calf serum
HSC: Hematopoietic stem cell
Ig: Immunoglobulin
IL: Interleukin
M-CSF: Macrophage colony-stimulating factor
NA: Noradrenaline
NaCl: Sodium chloride
NGS: Normal goat serum
p. i.: Post-immunization
PACAP: Pituitary adenylate cyclase-activating peptide
PBMC: Peripheral blood mononuclear cell
PBS: Phosphate-buffered saline
PE: Phycoerythrin
RA: Rheumatoid arthritis
RT: Room temperature
TH: Tyrosine hydroxylase
TNF: Tumor necrosis factor
VIP: Vasoactive intestinal peptide
α-MEM: Alpha-minimum essential medium
