# Supplementary material for: Reactivity of rat bone marrow-derived macrophages to neurotransmitter stimulation in the context of collagen II-induced arthritis
Source: Arthritis Res Ther. 2015 Jun 24;17(1):169. doi: 10.1186/s13075-015-0684-4 (PMC4496866; doi:10.1186/s13075-015-0684-4)
Supplement: Additional file 1: Table S1. — Primer sequences for endpoint polymerase chain reaction (PCR). bp base pairs. [file 13075_2015_684_MOESM1_ESM.docx]

**Additional file 1: Table S1: Primer sequences for Endpoint PCR**

| **Gene name** | **Primer sequence [5‘🡪3‘]** | **Tm [°C]** | | **Amplicon length [bp]** | | **Acc.No.** |
| --- | --- | --- | --- | --- | --- | --- |
| VIP receptor 1 | Fwd: CCTCCCTGTGGTGGATCATA | 58,2 | 60 bp | | NM_012685.2 | |
|  | Rev: GGACGAAGTTCACCAGGATG | 58,3 |  | |  | |
| VIP receptor 2 | Fwd: CAGATGTTGGTGGCAATGAC | 57,4 | 165 bp | | NM_017238 | |
|  | Rev: CCTGGAAGGAACCAACACAT | 57,7 |  | |  | |
| PACAP receptor 1 | Fwd: ACGTCAGCAAGAGGGAAAGA | 59,0 | 136 bp | | NM_133511 | |
|  | Rev: ACCTTCCAGCTCCTCCATTT | 58,6 |  | |  | |
| M1 muscarinic AChR | Fwd: ACAGCTGGCCAAGAGAAAGA | 59,2 | 180 bp | | NM_080773 | |
|  | Rev: CATAGCCAGTAGCCCAGCTC | 60,0 |  | |  | |
| M2 muscarinic AChR | Fwd: GGTCAGAGTGGGGATGAAAA | 57,1 | 135 bp | | NM_031016 | |
|  | Rev: AGCCAACAGGATAGCCAAGA | 58,7 |  | |  | |
| M3 muscarinic AChR | Fwd: ACAGCTGCATACCCAAAACC | 58,7 | 205 bp | | NM_012527 | |
|  | Rev: GGCACTCGCTTGTGAAAAAT | 57,6 |  | |  | |
| M4 muscarinic AChR | Fwd: CAGAGCTGTATCCCCGAGAG | 59,0 | 104 bp | | NM_031547.1 | |
|  | Rev: TTGAAAGTGGCATTGCAGAG | 56,9 |  | |  | |
| M5 muscarinic AChR | Fwd: CAG AGA AGC GAA CCA AGG AC | 59,4 | 236 bp | | NM_017362.4 | |
|  | Rev: CTC AGC CTT TTC CCA GTC AG | 59,4 |  | |  | |
| α7 nicotinic AChR | Fwd: GCTCCTGCTACATTGACGTTC | 59,8 | 105 bp | | NM_012832.3 | |
|  | Rev: GCATTTGCAGGTCCAGTGAC | 59,4 |  | |  | |
| adrenoceptor α1A | Fwd: TGATCCTCTCAGTGGCCTGTC | 61,2 | 101 bp | | NM_017191 | |
|  | Rev: CAGCACAGTGGAGGTGAGGAG | 62,4 |  | |  | |
| adrenoceptor α1B | Fwd: AACCTTGGGCATTGTAGTCG | 58,2 | 147 bp | | NM_016991 | |
|  | Rev: AGGCAGCTGTTGAAGTAGCC | 60,3 |  | |  | |
| adrenoceptor α1D | Fwd: CAA CCT GCT GGT CAT CCT TT | 58,1 | 233 bp | | NM_024483.1 | |
|  | Rev: AGATGGTGCAGAGGCTAAGG | 59,2 |  | |  | |
| adrenoceptor α2A | Fwd: GTGTGTTGGTTCCCGTTCTT | 58,6 | 149 bp | | NM_012739 | |
|  | Rev: CGGAAGTCGTGGTTGAAAAT | 56,7 |  | |  | |
| adrenoceptor α2B | Fwd: AGTTTTCGTGGTCTGCTGGT | 58,8 | 163 bp | | NM_138505.2 | |
|  | Rev: GTCCTGGTTGAAGACGGTGT | 59,9 |  | |  | |
| adrenoceptor α2C | Fwd: TCCGTCGAGTTCTTCCTGTC | 59,1 | 141 bp | | NM_138506 | |
|  | Rev: GAAGGGGAACCAGCACAGTA | 59,3 |  | |  | |
| adrenoceptor β1 | Fwd: TCAAGACACTGGGCATCATC | 57,6 | 130 bp | | NM_012701 | |
|  | Rev: CCCAGCCAGTTGAAGAAGAC | 58,5 |  | |  | |
| adrenoceptor β2 | Fwd: CACATCGCCCTTCAAGTACC | 59,6 | 116 bp | | NM_012492 | |
|  | Rev: ACCAGTGCATCTGGATAGGC | 59,5 |  | |  | |
| adrenoceptor β3 | Fwd: CGCACCTTGGGTCTCATTAT | 57,7 | 150 bp | | NM_013108 | |
|  | Rev: GAAGGCAGAGTTGGCATAGC | 59,0 |  | |  | |
