# Supplementary figures and images for: Reactivity of rat bone marrow-derived macrophages to neurotransmitter stimulation in the context of collagen II-induced arthritis
Source: Arthritis Res Ther. 2015 Jun 24;17(1):169. doi: 10.1186/s13075-015-0684-4 (PMC4496866; doi:10.1186/s13075-015-0684-4)

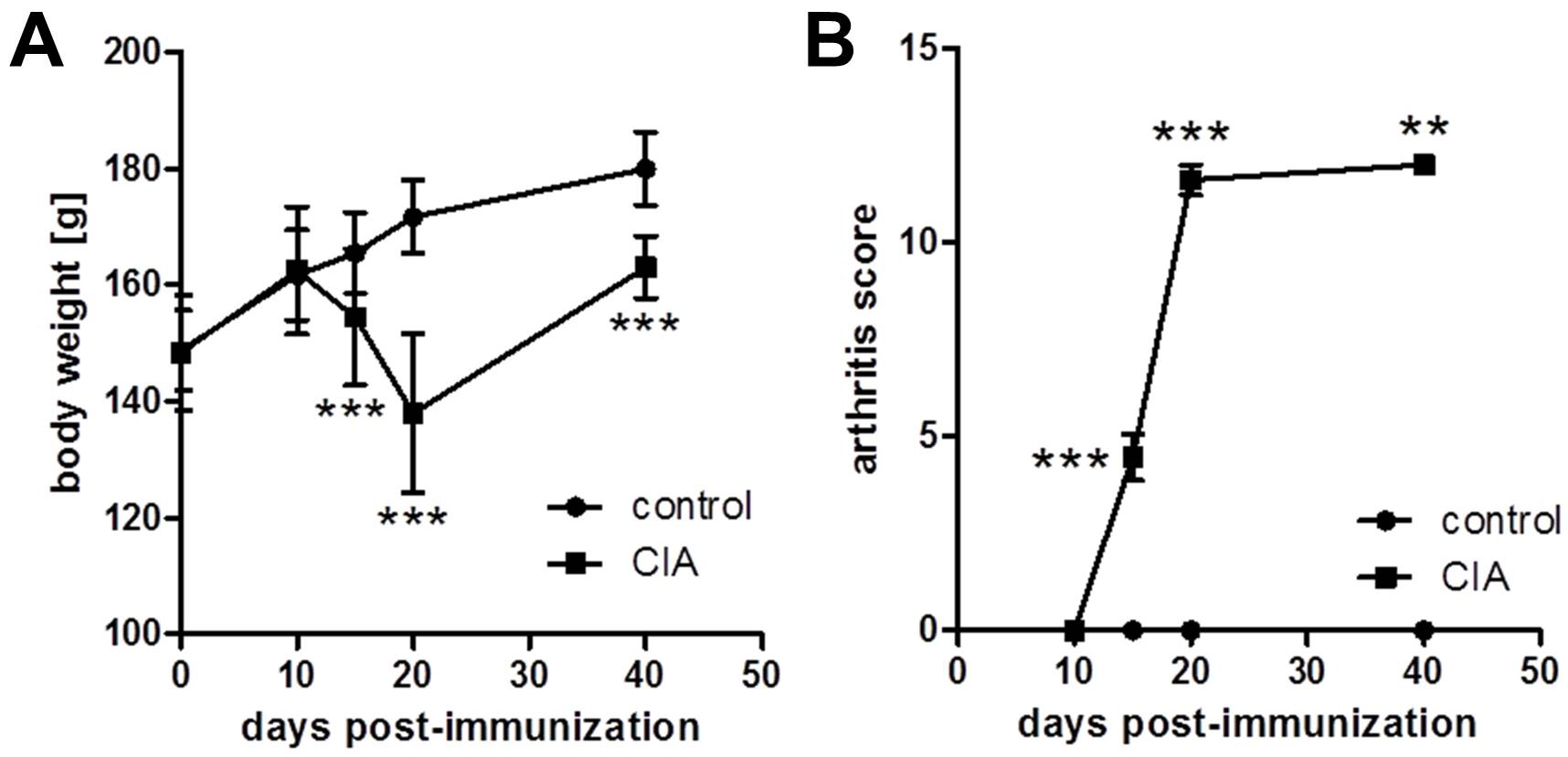

Supplement: Additional file 2: Figure S1. — Collagen type II-induced arthritis (CIA). Successful CIA induction was verified by monitoring body weight (a) and arthritis score (b) of arthritic and control animals 10 (n = 40), 15 (n = 30), 20 (n = 20), and 40 (n = 10) days post immunization. Data are expressed as mean ± standard error of the mean. **P < 0.01, ***P < 0.001. [file 13075_2015_684_MOESM2_ESM.jpeg]

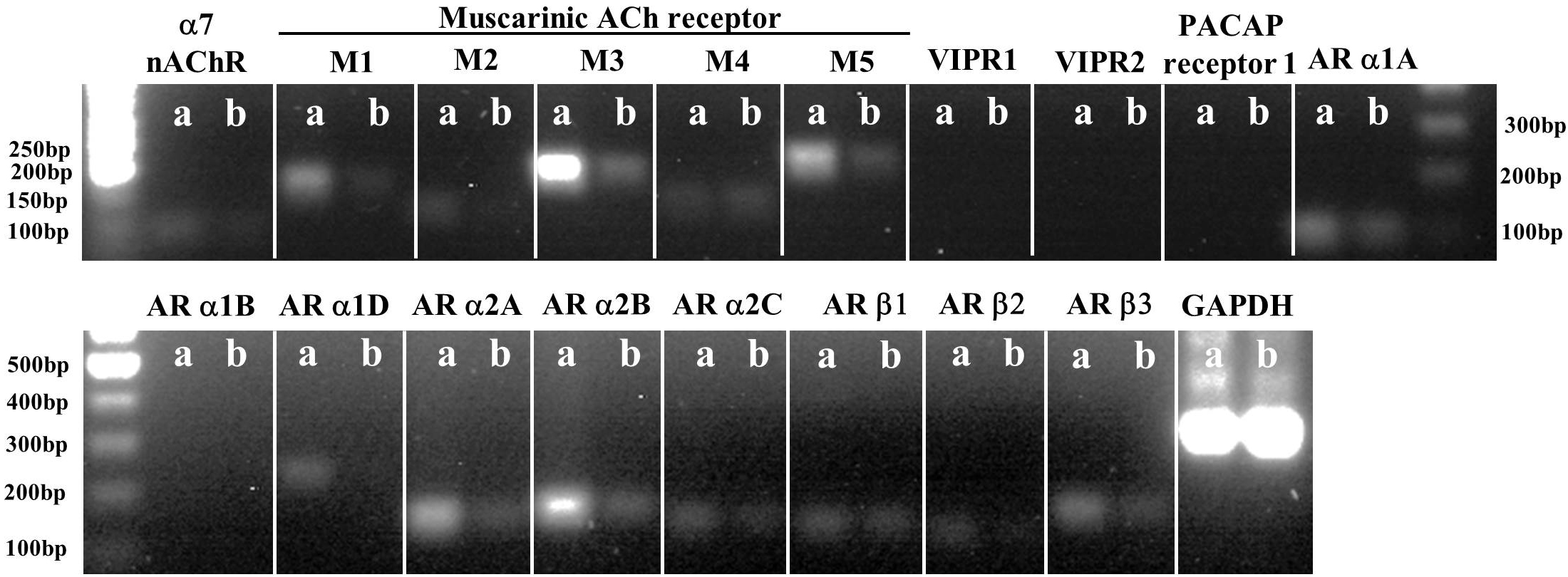

Supplement: Additional file 3: Figure S2. — Endpoint polymerase chain reaction for neurotransmitter receptors. Gene expression of neurotransmitter receptors for ACh, NA, and VIP was analyzed by endpoint polymerase chain reaction by using RNA isolated from BMM of controls 10 days (a) and 20 days (b) following sodium chloride treatment. N = 1. AR adrenoceptors, BMM bone marrow-derived macrophage, bp base pairs, M1-M5 muscarinic acetylcholine receptor, NA noradrenaline, nAChR nicotinic acetylcholine receptor, PACAPR1 pituitary adenylate cyclase-activating peptide receptor 1, VIPR1-2 vasoactive intestinal peptide receptors 1 and 2. [file 13075_2015_684_MOESM3_ESM.jpeg]

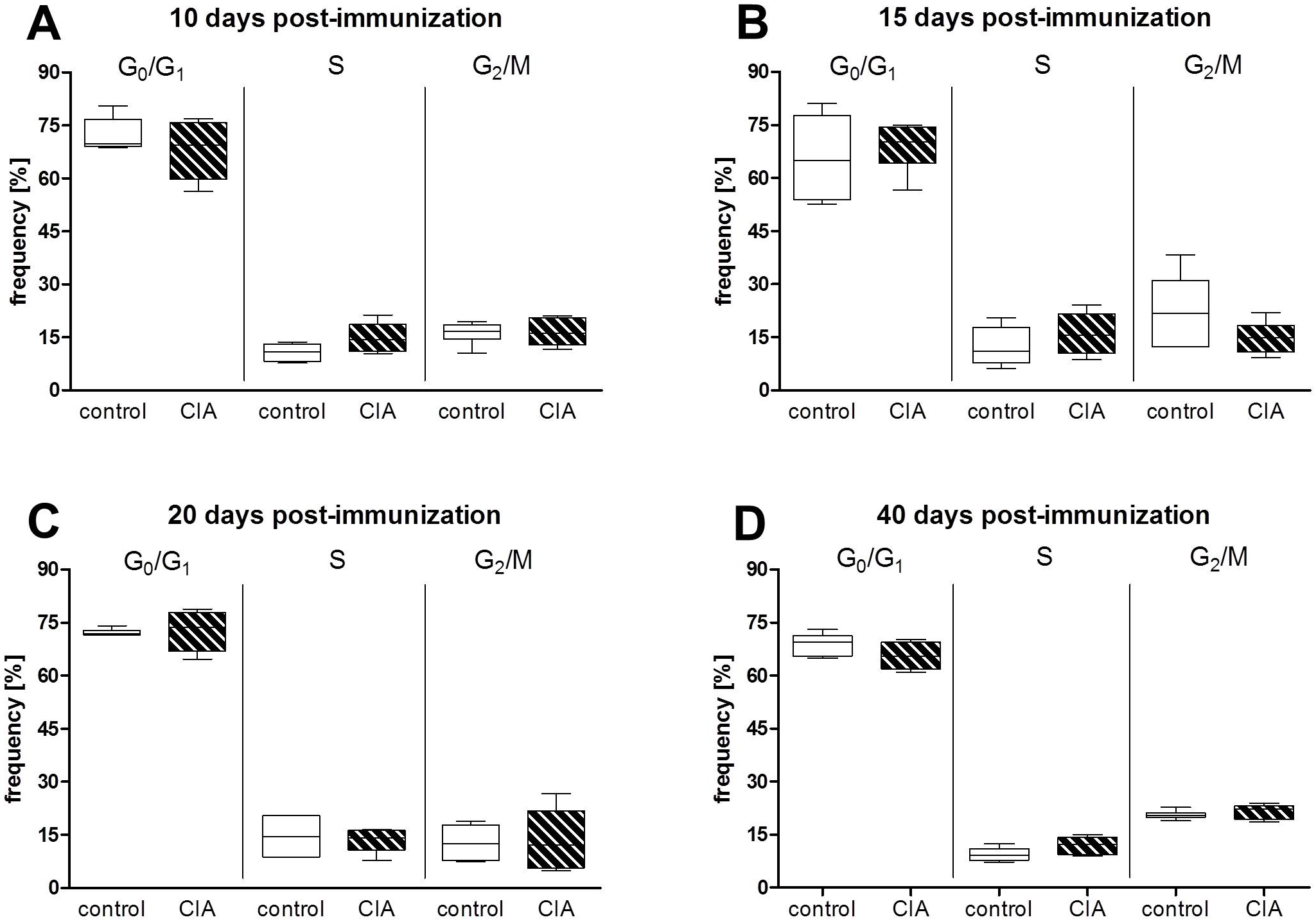

Supplement: Additional file 5: Figure S3. — Cell cycle analysis. G0/G1 phase BMMs from control and CIA rats were cultured in the presence of fetal calf serum and macrophage colony-stimulating factor for 48 h and cell cycle distribution was analyzed by measurement of nucleic propidium iodide content. The graph compares the frequency of BMMs from control and CIA animals distributed in cell cycle phases G0/G1, S, and G2/M. Results for 10 days p. i. are presented under (a), 15 days p. i. under (b), 20 days p. i. under (c) and 40 days p. i. under (d). N = 6 for control and CIA at each time point. BMM bone marrow-derived macrophage, CIA collagen II-induced arthritis, p. i. post-immunization. [file 13075_2015_684_MOESM5_ESM.jpeg]
