# Supplementary material for: Reactivity of rat bone marrow-derived macrophages to neurotransmitter stimulation in the context of collagen II-induced arthritis
Source: Arthritis Res Ther. 2015 Jun 24;17(1):169. doi: 10.1186/s13075-015-0684-4 (PMC4496866; doi:10.1186/s13075-015-0684-4)
Supplement: Additional file 4: Table S2. — Data summary for adhesion, apoptosis, and proliferation assay. Table S2 summarizes data for adhesion assay (a-d), caspase 3/7 assay (e), and proliferation assay (f). Non-stimulated CIA data are shown as percentage to BMM from control (100 %). Effects of neurotransmitter stimulation are presented as percentage to the respective non-stimulated CIA and control BMM (100 %). Data are expressed as mean ± standard error of the mean. *P < 0.05 neurotransmitter stimulation versus non-stimulated cells; # P < 0.05; ## P < 0.01; ### P < 0.001 control cells versus CIA cells. ACh acetylcholine, BMM bone marrow-derived macrophage, CIA collagen II-induced arthritis, NA noradrenaline, VIP vasoactive intestinal peptide. [file 13075_2015_684_MOESM4_ESM.docx]

**Additional file 4: Table S2: Data summary for adhesion, apoptosis and proliferation assay**

|  | **10 days p.I.** | | **15 days p.I.** | | **20 days p.I.** | | **40 days p.I.** | |
| --- | --- | --- | --- | --- | --- | --- | --- | --- |
|  | **control** | **CIA** | **control** | **CIA** | **control** | **CIA** | **control** | **CIA** |
| **A) Adhesion assay [%] - Plastic** | | | | | | | | |
| non-stimulated | 100±7.9 | -15.3±15.8 | 100±5.1 | -10.1±5.2 | 100±3.7 | ^##^-44.5±2.9 | 100±11.9 | -21.2±12.3 |
| ACh 10^-6^M | +2.4±6.8 | -5.7±5.5 | +4.3±4.5 | *^,#^-16.5±5.9 | +14.7±5.9 | +21.5±8.1 | +2.3±3.3 | +9.5±13.1 |
| VIP 10^-9^M | -5.1±5.6 | -14.4±3.6 | -2.1±8.0 | -8.3±3.4 | +20±8.6 | ^*^+41.3±10.2 | +0.9±6.4 | ^*^+29.6±13.0 |
| NA 10^-6^M | ^*^-13±5.3 | -14.1±5.4 | -2.6±2.9 | -19.8±9.6 | +10.6±3.3 | +20.4±9.0 | +4.5±3.2 | -0.8±8.5 |
| NA 10^-8^M | -6.1±8.7 | +0.2±5.3 | +5.6±6.9 | -23.5±8.4 | +20.8±5.2 | +20.4±9.1 | ^*^+19.5±7.7 | +8.8±8.6 |
| **B) Adhesion assay [%] – Collagen I** | | | | | | | | |
| non-stimulated |  |  |  |  | 100±23.8 | -39.5 ± 9.9 | 100±5.7 | ^##^-52.3 ± 8.5 |
| ACh 10^-6^M |  |  |  |  | +75.7 ± 63.6 | +8.0 ± 23.5 | -2.1 ± 5.6 | +39.6 ± 23.5 |
| VIP 10^-9^M |  |  |  |  | -25.0 ± 17.5 | +2.0 ± 34.5 | -6.2 ± 12.7 | -17.6 ± 12.7 |
| NA 10^-6^M |  |  |  |  | -34.2 ± 5.05 | -29.4 ± 20.0 | ^*^-37.8 ± 7.7 | -2.8 ± 20.5 |
| NA 10^-8^M |  |  |  |  | -28.4 ± 16.0 | -16.1 ± 18.3 | -7.0 ± 10.1 | +62.1 ± 67.1 |
| **C) Adhesion assay [%] – Fibronectin** | | | | | | | | |
| non-stimulated |  |  |  |  | 100±16.4 | -5.9 ± 23.4 | 100±4.7 | ^##^-32.7 ± 8.9 |
| ACh 10^-6^M |  |  |  |  | +10.0 ± 18.6 | +83.4 ± 52.5 | +6.9 ± 7.2 | -6.9 ± 6.3 |
| VIP 10^-9^M |  |  |  |  | +8.8 ± 32.5 | +87.3 ± 47.4 | -10.8 ± 7.0 | -18.0 ± 16.0 |
| NA 10^-6^M |  |  |  |  | -11.5 ± 26.7 | -17.9 ± 18.0 | -13.5 ± 9.0 | -20.6 ± 8.4 |
| NA 10^-8^M |  |  |  |  | +24.5 ± 38.6 | +60.1 ± 68.5 | -6.5 ± 8.9 | -12.6 ± 20.8 |
| **D) Adhesion assay [%] – Laminin** | | | | | | | | |
| non-stimulated |  |  |  |  | 100±7.4 | +15.5 ± 16.2 | 100±13.9 | -26.7 ± 15.1 |
| ACh 10^-6^M |  |  |  |  | +9.2 ± 17.6 | -5.9 ± 2.9 | +6.9 ± 18.8 | +11.0 ± 23.6 |
| VIP 10^-9^M |  |  |  |  | +23.0 ± 27.7 | 13.0 ± 14.7 | -12.7 ± 12.0 | +85.6 ± 60.6 |
| NA 10^-6^M |  |  |  |  | -22.1 ± 15.3 | -35.6 ± 9.7 | ^*^-37.4 ± 10.9 | +25.2 ± 29.0 |
| NA 10^-8^M |  |  |  |  | +19.1 ± 22.6 | -4.2 ± 55.5 | -2.9 ± 13.1 | +32.2 ± 43.1 |
| **E) Caspase 3/7 apoptosis assay [%]** | | | | | | | | |
| non-stimulated | 100±8.1 | -27.3±11.3 | 100±7.6 | +17.1±26.3 | 100±3.5 | +10.2±15.0 | 100±3.3 | +48.0±21.6 |
| ACh 10^-6^M | -3.6±10.4 | -8.5±7.5 | +139.8±56.6 | ^#^-56.9±6.6 | -15.5±8.4 | -7.1±8.6 | +7.5±17.7 | +3.0±7.2 |
| VIP 10^-9^M | -4.7±11.7 | -4.1±11.5 | +97.7±42.3 | ^#^-64.7±5.8 | ^*^-34.0±5.7 | +5.3±13.4 | +38.6±23.5 | -8.5±11.8 |
| NA 10^-6^M | -4.9±13.6 | -12.1±8.8 | ^*^+172.9±68.0 | ^#^-66.4±5.8 | -23.4±9.2 | -14.1±8.8 | -4.6±17.1 | +3.0±9.9 |
| NA 10^-8^M | +4.7±12.2 | -4.5±7.6 | ^*^+164.0±63.8 | ^#^-54.4±6.4 | -19.6±10.3 | -1.3±12.7 | +18.9±11.1 | -3.6±7.5 |
| **F) BrdU proliferation assay [%]** | | | | | | | | |
| non-stimulated | 100±5.6 | -17.9±7.7 | 100±4.8 | ^###^-41.2±4.3 | 100±5.3 | +0.6±9.3 | 100±4.8 | ^##^-33.4±7.9 |
| ACh 10^-6^M | +9.6±6.9 | +2.0±10.2 | -5.0±12.8 | +2.0±10.1 | ^*^+35.6±13.7 | ^#^-12.1±8.8 | ^*^-29.9±6.9 | ^*^-34.7±7.3 |
| VIP 10^-9^M | -12.3±7.5 | -10±10.0 | -17.7±19.7 | +26.8±23.4 | ^*^+58.9±23.6 | ^#^-28.5±14.2 | ^*^-41.9±7.1 | -31.6±14.8 |
| NA 10^-6^M | -1.4±4.2 | -22.7±11 | -27.4±15.8 | -2.2±14.2 | +13.6±18.5 | -15.1±7.0 | ^*^-49.2±7.9 | ^*^-23.8±7.5 |
| NA 10^-8^M | +11.4±6.3 | +1.5±10.9 | -30.9±13.1 | -1.8±16.7 | +23.4±23.5 | -10.3±11.4 | ^*^-51.0±5.2 | -11.2±17.2 |

Non-stimulated CIA data are shown as percentage to BMM from control (100%). Effects of neurotransmitter stimulation are presented as ±- percentage to the respective non-stimulated CIA and control BMM (100%). Data are expressed as mean ± SEM.

*p<0,05 NT stimulation vs. non-stimulated cells; #p<0,05; ##p<0,01; ###p<0,001 control cells vs. CIA cells.

CIA: collagen-induced arthritis, BMM: bone marrow macrophages, ACh: acetylcholine, VIP: vasoactive intestinal peptide, NA: noradrenaline
